# Aptamer-Functionalized Nanoparticles in Targeted Delivery and Cancer Therapy

**DOI:** 10.3390/ijms21239123

**Published:** 2020-11-30

**Authors:** Zhaoying Fu, Jim Xiang

**Affiliations:** 1 Institute of Molecular Biology and Immunology, College of Medicine, Yanan University, Yanan 716000, China; 2 Division of Oncology, University of Saskatchewan, Saskatoon, SK S7N 4H4, Canada

**Keywords:** aptamer, nanoparticle, delivery, cancer

## Abstract

Using nanoparticles to carry and delivery anticancer drugs holds much promise in cancer therapy, but nanoparticles per se are lacking specificity. Active targeting, that is, using specific ligands to functionalize nanoparticles, is attracting much attention in recent years. Aptamers, with their several favorable features like high specificity and affinity, small size, very low immunogenicity, relatively low cost for production, and easiness to store, are one of the best candidates for the specific ligands of nanoparticle functionalization. This review discusses the benefits and challenges of using aptamers to functionalize nanoparticles for active targeting and especially presents nearly all of the published works that address the topic of using aptamers to functionalize nanoparticles for targeted drug delivery and cancer therapy.

## 1. Introduction

The ideal cancer therapeutics should be capable of exerting maximum destruction on cancer cells while being able to keep damage to healthy tissues at a minimum. Many anticancer drugs are toxic to cancer cells and healthy cells largely non-differentially, and the major reason that they cause more damage to cancer is because the cancer cells grow/divide more quickly. Besides, most anticancer drugs are in general evenly distributed throughout the body when administered systemically and the result is that only a very small fraction of the drugs reach the diseased site. Therefore, it is not surprising that selective delivery of anticancer drugs to cancer cells has long been a vigorous pursuit of cancer scientists.

Nanoparticles have the potential to encapsulate and transport anticancer drugs to tumor tissue more effectively [1]. However, nanoparticles per se do not have specificity to cancer cells; the fact that nanoparticles accumulate preferentially in cancer sites is basically due to the enhanced permeability and retention (EPR) effect of the tumor tissue [2]. On the other hand, if nanoparticles could be functionalized by ligands capable of recognizing cancer cells specifically, they will be able to target and deliver cargoes selectively to cancer cells and thus greatly increase the therapeutic index (increasing therapeutic efficacy while reducing toxicity). To date, a number of moieties have been studied to functionalize nanoparticles for specific targeting and aptamer is one of them [3]. 

This paper discusses aptamer-functionalized nanoparticles in targeted delivery for cancer therapy. It first compares passive and active targeting of nanoparticles, then describes the advantages of using aptamers to functionalize nanoparticles for active targeting, explains the strategies to conjugate aptamers to nanoparticles, and summarizes nearly all of the existing aptamer-functionalized nanoparticles used thus far to study targeted delivery to cancer cells. It finally briefly discusses the challenges facing active targeting. 

## 2. Passive vs. Active Targeting of Nanoparticles

Passive targeting of nanoparticles refers to the passive accumulation of nanoparticles in the tumor tissue, which is generally attributed to the enhanced permeability and retention effect. The concept of EPR was first introduced more than 30 years ago when Maeda and colleagues found that certain macromolecules accumulate preferentially in the tumor tissue [4]. EPR is mainly the result of leakiness of the discontinuous endothelium of angiogenic tumor vasculature combined with defective lymphatic drainage of the tumor matrix, which facilitates the extravasation and accumulation of nanoparticles in tumor. It has been shown that the number of nanoparticles accumulated in tumor tissue may be 10–200 times higher than in normal tissue as a result of EPR. The EPR effect is considered to be the primary element to improve the efficacy and safety of nanotherapeutics. In fact, most of the nanomedicines marketed thus far base their increased therapeutic index mainly on the EPR effect [5].

Nevertheless, the EPR effect alone is insufficient for adequate nanoparticle accumulation, particularly in some circumstances. The EPR effect is not effective for some cancers because of tumor heterogeneity and cancer stage, is even not applicable to some types of cancers, and it is not effective in some patients because of individual differences. A survey of the literature in this area from 2005 to 2015 that included 232 data sets showed that only a median of 0.7% of the systemically administered nanoparticle dose could reach the solid tumor in mouse models [6]; multivariate analysis of the pertinent parameters indicated that tumor type, tumor model, and nanomaterial properties are the major factors to affect the delivery efficiency of the nanoparticles. Research also found that the high interstitial fluid pressure of tumor tissue impedes the extravasation of nanoparticles [7]; some particles that have entered the tumor intercellular space via EPR effect may be forced back into the blood circulation because of the high fluid pressure within the tumor interstitium. It is manifest that blood cancers, very early stage tumors, and small metastasized cancers do not have or have only insignificant EPR effect. In addition, because of tumor heterogeneity, the EPR effect is very poor or not shown in some types of cancers and even in different regions of the same tumor [8]. Clinical observations have also indicated that the EPR effect exhibits significant individual variations among patients; the nanomedicines do not increase the therapeutic efficacy in some subpopulations of the patients [9]. Finally, and most importantly, it is now reckoned that the EPR effect chiefly works in animal models rather than in humans [10]; in patients, their effects are just uncertain (because of interpatient variability); these uncertainties pose the most serious challenge to the rationale of nanomedicine development based on the EPR effect and to the clinical translation of the nanotherapeutics. All the above problems warrant the development of a more effective way to deliver nanoparticles to the site of interest.

Active targeting, which is achieved by conjugating tumor specific ligands to the surface of nanoparticles, can provide a means to complement the EPR effect or solve the aforementioned problems. Common classes of targeting ligands that can functionalize nanoparticles include antibodies or antibody fragments, aptamers, carbohydrates, human transferrin protein, peptides, and vitamins such as folate, etc. Representative tumor biomarkers that can be recognized by the targeting ligands include epidermal growth factor receptor (EGFR), epithelial cell adhesion molecule (EpCAM), human epidermal growth factor receptor 2 (HER2), Mucin-1 (MUC1), nucleolin, platelet-derived growth factor receptor β (PGFRβ), prostate specific membrane antigen (PSMA), transferrin receptor, folate receptor, and so on.

The foremost advantage of actively targeted nanoparticles over passively targeted nanoparticles is that they can add on to or improve the EPR effect. An actively targeted nanoparticle can first enter the tumor tissue via the EPR effect and then target cancer cells through specific ligand recognition of the tumor biomarker. In addition, active targeting can augment the EPR effect by having more particles entering than leaving the tumor interstitium because the particles that already enter stick to the cancer cells and thus lower the concentration of the free nanoparticles in the interstitial space. Studies have already demonstrated that actively targeted nanoparticles tend to accumulate more efficiently in the tumor tissue through their selective binding to receptors on the cancer cells when they enter the tumor interstitium [11].

The ligand-mediated active targeting not only helps nanoparticles selectively reach the tumor; it may also promote cellular internalization of the nanoparticles through receptor-mediated endocytosis since some receptors have the intrinsic property to internalize when bound by a ligand. The importance of cellular internalization should be obvious when we think of the fact that most anticancer drugs exert their actions inside cancer cells. Although nanoparticles themselves can get into the cell through clathirin-mediated endocytosis or fluid-phase pinocytosis, conjugation of active ligands to them may boost the process. Receptor-mediated engulfment has already been observed in many specific ligand conjugated nanoparticles; typical examples of aptamer-mediated cellular internalization include the PSMA-targeting A10 aptamer mediated as well as the nucleolin-targeting AS1411 aptamer mediated internalizations [12,13].

Although the targeting ligands can be conjugated with the anticancer agents such as siRNAs and chemotherapeutics directly, the advantage of using nanoparticles is that they can deliver large amounts of drug payload or diversified therapeutics to cancer cells per delivery and biorecognition event [14]. Having a nanoparticle encapsulate diverse therapeutic ingredients could potentially offer synergistic tumor killing effects (e.g., combining any of these anticancer strategies like chemotherapy, gene silencing, immunotherapy, photodynamic therapy, photothermal therapy, and thermodynamic therapy, etc.). Encapsulating different therapeutics within a nanoparticle may also help to overcome or reduce multiple drug resistance (MDR) because MDR usually does not occur to different drugs at the same time or at the same degree, and the mechanisms of MDR differs with different drugs. One example is that nanoparticle-mediated combination of chemotherapy and photodynamic therapy can overcome drug resistance through invoking multiple anticancer mechanisms including cytotoxicity and significantly enhanced production of reactive oxygen species [15].

Active targeting of nanoparticles could also have additive therapeutic effects by exploiting the drug-carrying and receptor-inhibiting actions at the same time. For instance, anticancer reagent-containing nanoparticles functionalized with HER2-targeting ligand, in addition to delivering the therapeutic ingredients into the target cells can, meanwhile, inhibit the activity of the targeted receptors or remove the receptors from the cell surface by means of internalization [16].

## 3. Aptamer-Functionalized Nanoparticles in Actively Targeted Drug Delivery

Aptamers are short single-stranded DNA or RNA molecules with defined three-dimensional structures that can selectively bind to target molecules with high affinity [17]. Aptamers are usually produced by selecting them from a large random sequence pool with the technology systematic evolution of ligands by exponential enrichment (SELEX). In addition to their superb binding specificity and affinity, aptamers have a number of other favorable features that together make them very suitable molecules to functionalize nanoparticles for actively targeted delivery. Aptamer functionalized nanoparticles have already demonstrated their effectiveness in targeted delivery of anticancer drugs in numerous preclinical and animal studies, though none of them have as yet entered clinical trial or application.

### 3.1. The Advantages of Using Aptamers to Functionalize Nanoparticles

Aptamers have a very broad spectrum of target recognition and binding; they have little or no immunogenicity; they can easily be end-attached with a chemical group to conjugate nanoparticles; they are small (only a few nanometers in diameter) and will not increase nanoparticle size significantly after coupling; they are relatively easy to make and to store [17]. Those are the general properties of aptamers that make them one of the best choices to functionalize nanoparticles. Up to now, quite a few aptamers have been used to functionalize nanoparticles for targeted delivery to cancer cells (Table 1).

**Figure 1:**
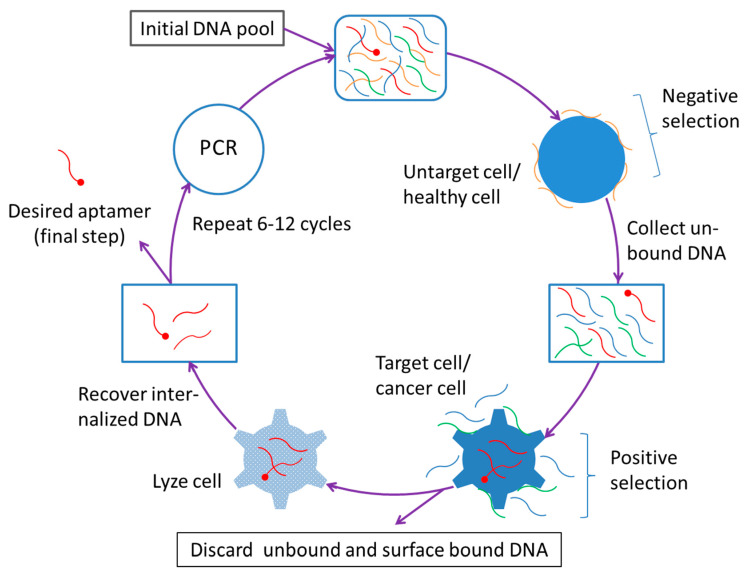
Selection procedure of cell-internalizing DNA aptamer using cell-SELEX.

Apart from the abovementioned characteristics, aptamers have a unique advantage that is related to their production—the establishment of the cell-SELEX technique and its improvements have made the aptamer an especially useful ligand to be used to construct the cancer-targeting nanocarriers (Figure 1).

After the setting up of the prototype SELEX technology in 1990, a selection strategy known as cell-SELEX was developed in 2003 that uses whole (living) cells to select aptamers targeting cell surface molecules [215]. This technique allows for the isolation of cell-recognizing aptamers without prior knowledge of the target molecule(s). In 2006, a negative selection (or counter-selection) process was integrated into the original cell-SELEX strategy, which makes it possible to obtain cell-specific aptamers on researcher’s will [216]. In the new cell-SELEX procedure, the negative selection is performed first, wherein the negative-selection cells (these may be normal cells or any untargeted cells and several different types of cells may be used) are used to absorb the undesired or non-specific aptamers (In this step, the undesired or non-specific oligonucleotides in the pool are removed as they bind to the negative-selection cells). The negative selection is followed by positive selection that is conducted basically in the same way as the conventional cell-SELEX strategy and aims to discard the oligonucleotides that do not bind to the positive-selection cells (usually, certain types of cancer cells or any researcher-intended cells are used for this purpose). Thus, by employing the new cell-SELEX technique, one is able to generate aptamers that can specifically recognize cell surface receptors (or molecules) and thus can effectively differentiate cancer cells from normal cells. More importantly, with certain added steps, the cell-SELEX technique can still select aptamers that not only specifically recognize or target cell surface receptors but also get into the cells through receptor mediated internalization [217].

### 3.2. Strategies of Conjugating Aptamers to Nanoparticles

**Figure 2:**
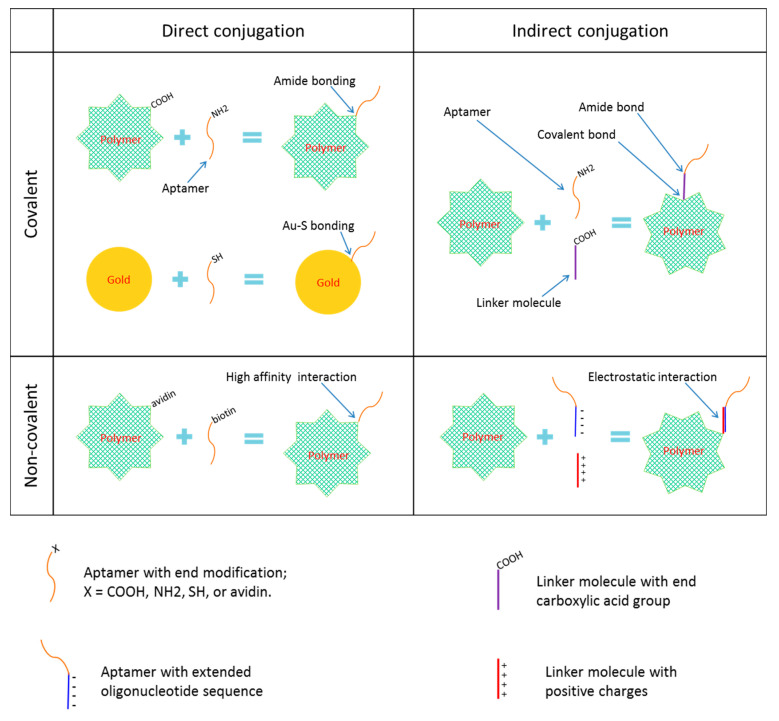
Common strategies of nanoparticle-aptamer conjugation.

Aptamers can be conjugated to nanoparticles directly or indirectly via a linker molecule (a bridge or spacer). Both direct and indirect conjugation can be achieved either covalently or non-covalently (Figure 2).

In covalent conjugation, a functional group (such as a primary amino group or a thiol group) is usually attached to one terminus of the aptamer, which can react with the functional group (such as the carboxylic acid group, the maleimide group, and the aldehyde group) on the surface of the nanoparticle or at one end of the linker molecule, or react with the gold or other metal element or inorganic molecule for inorganic nanoparticles. Common examples of covalent conjugation include the carboxylic acid group and the amino group interaction that results in an amide (or carboxamide) linkage, the carboxylic acid group and the thiol group interaction that results in a thioester bond, the carboxylic acid group and the alcohol group interaction that results in an ester bond, the primary amine group and thiol group interaction that results in a thioamide bond, the thiol group and the thiol group interaction that results in a disulfide bond, and the thiol group and the gold or silver interaction that results in a Au–S or Ag-S bond.

Non-covalent conjugation strategies include high affinity interactions and electrostatic interactions. The former includes avidin–biotin and streptavidin–biotin interactions. The latter are commonly seen when a linker molecule is used, in which case the opposite charges on the linker molecule and on the extended oligonucleotide sequence of the aptamer interact, but also include the using of histidine tags.

Most of the aptamer–nanoparticle conjugates reported thus far utilized the direct and covalent strategy. According to Farokhzad and colleagues [11], “covalently linked bioconjugates may result in enhanced stability in physiological salt and pH whilst avoiding the unnecessary addition of biological components (i.e., streptavidin); thus minimizing immunological reactions and potential toxicity”. Fewer studies used bridge or spacer molecule to link aptamer and nanoparticle together. These are in consideration of avoiding any possible steric or spatial restrictions on aptamer’s binding to target molecule, but an associated problem is the increased size of the conjugates. Several aptamer-nanoparticle constructions, including both direct and indirect linkage, used the avidin–biotin or the streptavidin–biotin system. These interactions are very stable but the bulk of the formulation may increase considerably and potential immunological rejection problems might also result.

### 3.3. Aptamer-Functionalized Nanoparticles in Pre-Clinical Studies

**Figure 3:**
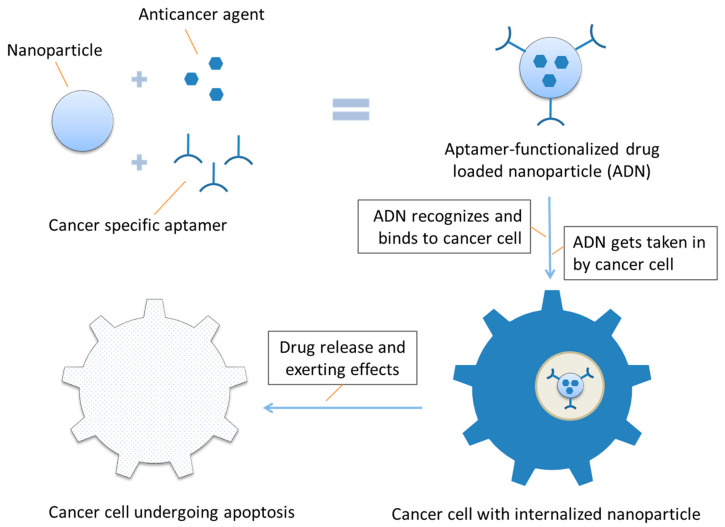
Schematic representation of aptamer-functionalized nanoparticle acting on a cancer cell.

Up till now, quite a lot of aptamer-conjugated nanoparticles have been developed that can target specific cancer cells, deliver various therapeutic agents into cancer cells, and result in cancer cell toxicity in vitro (e.g., inhibit cell proliferation and induce apoptosis of cultivated cancer cells) and/or anticancer effects in vivo (e.g., inhibit xenograft tumor formation in nude mouse model). An inclusive list of nearly all aptamer-conjugated drug-delivering nanoparticles that have been studied thus far with their characteristics and sources is provided in Table 1. A schematic representation of the action process of aptamer-functionalized nanoparticles acting on a cancer cell is shown in Figure 3.

Farokhzad and Langer et al. [18] first performed the proof of concept study of using the aptamer to functionalize nanoparticles for actively targeted drug delivery in 2004. The authors synthesized the nanoparticles of poly (lactic acid)-block-polyethylene glycol copolymer with a terminal carboxylic acid functional group (PLA-b-PEG-COOH) and encapsulated the nanoparticles with rhodamine-labeled dextran as a model drug; they then covalently attached the PSMA-targeting A10 RNA aptamer to the nanoparticles through the reaction of the amino groups on the 3′ end of the aptamers with the carboxylic acid groups on the surface of the nanoparticles. These aptamer–nanoparticle conjugates were demonstrated to be able to target the PSMA-positive prostate LNCaP cells significantly more efficiently compared with the same PEGylated nanoparticles without aptamer conjugation and could get internalized into the cells. The uptake of these conjugates was not boosted in the PC3 cells that are also prostate-derived but do not express PSMA.

A similar nanoparticle-aptamer construction, which used the same PSMA-targeting aptamer but used poly (lactic-co-glycolic acid)-block-polyethylene glycol copolymer with a terminal carboxylic acid group (PLGA-b-PEG-COOH) as nanomaterial and encapsulated the anticancer drug Docetaxel within the nanoparticles, was later assessed both in vitro and in vivo by the same laboratory. The in vivo results showed that the aptamer-targeted drug-loaded nanoparticles exhibited significantly more reduced toxicity (side effects) in the nude mice as measured by mean body weight loss than non-targeted nanoparticles, and intratumoral injection of these aptamer-targeted drug-loaded nanoparticles resulted in complete tumor reduction in five of seven LNCaP xenograft nude mice compared with two of five for non-targeted nanoparticles [19].

Up to the present time, polymers, which include miscellaneous classes with PLGA-PEG being the most frequently used, remain the most used nanomaterials to construct aptamer functionalized nanoparticles to study targeted delivery for cancer therapy, followed by lipid based materials, particularly liposomes and nucleic acid based nanoparticles, including either DNA or RNA. Other organic nanomaterials that have been used include dendrimers, chitosan, proteins/peptides, or hybrids of the above. There are also many inorganic nanomaterials that have been studied in this area, including gold (Au) compounds, silver (Ag), mesoporous silica, graphene based, Calcium carbonate, ZnO, iron, etc. Other and special inorganic nanomaterials include magnetic nanomaterials, quantum dot based nanoparticles, and so on. In addition, organic and inorganic hybrids have also been used. Refer to Table 2 for a classified list of these nanoparticles and nanomaterials with their payloads, targets, related cancers, etc.

## 4. Challenges Facing Actively Targeted Delivery

Although active targeting holds much promise, several challenges exist. These include the increased complexity of synthesis and purification, the increased cost to make the conjugants, the alterations of nanoparticle properties, choosing a suitable tumor marker or receptor to target, and so forth.

### 4.1. Potential Alterations of Nanoparticle and Ligand Properties after Conjugation

Ligand conjugation may alter the properties of the nanoparticle. Not only will it increase nanoparticle size; it can also change the charge and modify the conformation of the nanoparticle. The change of nanoparticle size is likely to affect their pharmacokinetics; the change of nanoparticle charge will probably complicate their cellular uptake; the change of nanoparticle conformation may influence the binding feature of the attached ligand because of inadequate steric freedom or decreased orientation. All these must be taken into consideration in making actively targeting nanoparticles.

Although conjugating ligands to nanoparticles might change the pharmacokinetic property of the nanoparticles, this may not be a problem for aptamer conjugation because aptamers are very small, about 2–3 nm in length, in comparison with the drug-carrying nanoparticles, which is typically around 100 nm or larger in diameter. In fact, no literature has reported any alterations in the pharmacokinetics of nanoparticles following aptamer coupling.

Aptamers are commonly modified before therapeutic use. The purpose of modification is to increase their stability against nuclease degradation or prolong their half-life against kidney filtration. Aptamer modification can be performed during selection or after selection. The former aims at stabilizing the aptamers against nucleases. The latter aims at prolonging renal retention and is frequently done with PEGylation, covalent attachment of PEG to one end of the aptamer. Therefore, the attachment of aptamers to a nanoparticle will favorably increase their stability.

However, conjugation of aptamers to a nanoparticle might interfere with their proper folding and change their binding specificity and affinity. For example, the surface charge of the nanoparticle and the density of the attached aptamers on the nanoparticle may both affect their folding and three-dimensional structure. In addition, aptamers that are coupled directly to a nanoparticle may not recognize and bind their target effectively because there is no sufficient space (stereo-interference effect). Sometimes, the orientation of aptamer immobilization may also affect aptamer binding. All these problems should be considered by the researchers and optimum parameters or corresponding resolving measures be taken. For instance, the density and the orientation of attached aptamers can be investigated and optimized, and when stereo-interference occurs, the researchers can consider the use of a spacer molecule.

### 4.2. Selection of Suitable Tumor Marker or Receptor

The ideal receptor for targeted therapy is one that is exclusively presented on the tumor cells but not on the healthy cells. However, such a receptor may not exist in reality. What we can do is to choose the receptors that have a higher expression level on tumor cells than on healthy cells. The expression of the target receptors on healthy cells, though at a lower level, still carries a potential risk of off target binding. What is more, binding to these receptors may consume or waste the therapeutic nanoparticles and lower its concentration to reach the tumor.

### 4.3. The “Binding Site Barrier” Effect

Aside from the challenges mentioned above, there may also be the “binding site barrier” problem, which refers to a situation wherein high affinity binding to target cells prevents in-depth and uniform penetration of the targeted therapeutics into the tumor tissue. This phenomenon was first observed by Weinstein and colleagues [218,219] with antibodies, which showed that (1) antibody–antigen binding in tumor-retarded antibody percolation and (2) high antibody affinity had a tendency to decrease antibody percolation. The explanation to the phenomenon that higher-affinity antibodies penetrate the tumor tissue less efficiently than lower-affinity antibodies is that during tissue penetration, the higher-affinity antibodies bind tightly to the cells they first meet and so there are fewer free antibody molecules available; in contrast, lower-affinity antibodies tend to bypass these target cells and can penetrate deeper. Although the “binding site barrier” was originally demonstrated in antigen–antibody interaction, it may be reasonably extrapolated to the actively targeted nanoparticles and a similar phenomenon has in fact be observed by Miao et al. [220] using anisamide ligand targeted lipid-coated calcium phosphate nanoparticles. Therefore, it is essential to seek a balance between the affinity of active tumor targeting and the depth of nanoparticle penetration; trial and error may be necessary [221].

## 5. Conclusions

The first nanotechnology-based anticancer medicine was approved by the United States Food and Drug Administration (FDA) in 1996, which used PEGylated liposomes to encapsulate the chemotherapeutic drug doxorubicin. Today, about ten nanoparticle based medications are on the market (approved by FDA or other agencies) for cancer therapy [14,222]. All of them are non-targeted or passively targeted. These nanodrugs could delay the clearance or prolong the half-life of the drugs and reduce side-effects to a certain degree. However, only a modest increase in therapeutic efficacy could be observed and the undesired off-target problem still exists, which calls for the development of active targeting of nanoparticles. At the present time, more than a dozen nanoparticles for cancer therapy are undergoing clinical trials [2], of which several are actively targeted, but none of them are aptamer-functionalized. Actively targeted, especially aptamer-functionalized, nanoparticles hold great promise for future nanodrug development and applications. Therefore, more efforts are needed to further the investigation in this area, to refine the experiments and overcome the obstacles for clinical translation. Some obstacles for developing aptamer conjugated-nanoparticles into clinical use include insufficient data about their off-target effects and toxicity either in animals or in human. Venditto and Szoka once notified in their review paper titled *Cancer nanomedicines: so many papers and so few drugs* published in 2013 that “if we are truly interested in bringing more drugs into the clinic we should focus less on our publication record and more on devising scientific progress that translates into patient treatment” [223]. The same situation exists in the investigation of aptamer-functionalized nanoparticles when we take notice of the fact that more than two hundred papers have been published so far but none of the aptamer-functionalized nanoparticles have entered clinical trials, not to mention clinical application.

## Figures and Tables

**Table 1:** Aptamer-functionalized nanoparticles designed for actively targeted drug delivery and cancer therapy in laboratory investigation stage.

Aptamer	Nanomaterial	Payload	Conjugation	Size (nm)	Target	Cancer/Cell Line	Level	Ref.
A10, RNA	PLA-PEG-COOH	Rho-labeled dextran	Direct ^#^, covalent	≈264	PSMA	Prostate cancer	in vitro	[18]
A10, RNA	PLGA-PEG-COOH	Docetaxel	Direct, covalent	≈168	PSMA	Prostate cancer	in vitro + in vivo	[19]
A10, RNA	PLGA-PEG-COOH	Cisplatin	Direct, covalent	≈155	PSMA	Prostate cancer	in vitro	[20]
sgc8c, DNA	Au-Ag nanorod	Photothermal therapy	Direct, thiol linkage	No data	CCRF-CEM cell	ALL	in vitro	[21]
A10, RNA	SPION	Doxorubicin	Direct, covalent	66.4 ± 1.5	PSMA	LNCaP cell line	in vitro	[22]
sgc8c, DNA	PAMAM dendrimer	None	Direct, covalent	≈8	CCRF-CEM cell	ALL	in vitro	[23]
AS1411, DNA	Liposome	Cisplatin	Covalent, to cholesterol	≈200	Nucleolin	MCF-7 cells	in vitro	[24]
S2.2, DNA	PLGA-COOH	Paclitaxel	Covalent, DNA spacer	≈225.3	Mucin-1	Breast cancer	in vitro	[25]
AS1411, DNA	PEG-PLGA	Paclitaxel	Direct, covalent	156 ± 54.8	Nucleolin	Glioma	in vitro + in vivo	[26]
No name, DNA	DNA icosahedra	Doxorubicin	Direct, covalent	28.6 ± 5.0	Mucin-1	MCF-7 cells	in vitro	[27]
A9, RNA	SPION	Doxorubicin	ONT linker, base pairing	65 ± 12	PSMA	LNCaP cell line	in vitro + in vivo	[28]
A9, RNA	ONT-PAMAM dendrimer	Doxorubicin	ONT linker, base pairing	No data	PSMA	Prostate cancer	in vitro + in vivo	[29]
No name, RNA	QD-PMAT-PEI	siRNA	Chimera with siRNA	66.3–76.5	PSMA	C4–2B cells	in vitro	[30]
XEO2mini, RNA	Hybrid lipid-polymer	Docetaxel	Direct, covalent	50–100	PC3 cells	Prostate cancer	in vitro	[31]
AS1411, DNA	PLGA-lecithin-PEG	Paclitaxel	Covalent, to PEG	60–110	Nucleolin	GI-1 and MCF-7 cells	in vitro	[32]
AS1411, DNA	PLGA	Paclitaxel	Direct, amide linking	≈200	Nucleolin	GI-1 cells	in vitro	[33]
AS1411, DNA	PEG-PCL	Docetaxel, DiR, coumarin-6	Direct, covalent	170.6	Nucleolin	bEnd.3 and C6 cells	in vitro + in vivo	[34]
AS1411, DNA	Mesoporous silica	Gold nanorods *	ONT linker, base pairing	≈60	Nucleolin	MCF-7 cells	in vitro	[35]
GMT8, DNA	PEG-PCL	Docetaxel	Direct, covalent	111.9 ± 64.2	U87 cells	glioblastoma	in vitro + in vivo	[36]
AS1411, DNA	Gd:SrHap nanorod	Doxorubicin	Direct, covalent	153	Nucleolin	MCF-7 cells	in vitro	[37]
AS1411, DNA	Mesoporous silica	Doxorubicin	Electrostatic binding	≈140	Nucleolin	MCF-7 cells	in vitro	[38]
AS1411, DNA	Mesoporous silica	Fluorescein	Sulfo-GMBS linker	190	Nucleolin	MDA-MB-231	in vitro	[39]
Sgc8, DNA	Mesoporous silica	Doxorubicin	Avidin-biotin interaction	≈150	PTK7	CEM cells	in vitro	[40]
AS1411, DNA	Liposome	Doxorubicin	Covalent, to cholesterol	≈200	Nucleolin	MCF-7 breast cancer cells	in vitro + in vivo	[41]
A10, RNA	H40-PLA-PEG	Doxorubicin	Covalent, to PEG	≈69	PSMA	CWR22Rν1 cells	in vitro + in vivo	[42]
5TR1, DNA	SPION	Epirubicin	Direct, covalent	≈57	Mucin-1	carcinoma C26 cells	in vitro	[43]
No name, RNA	Hollow gold nanosphere	Doxorubicin	Direct, thiol–Au bonds	≈42	CD30	Lymphoma	in vitro	[44]
sgc8c, DNA	Aptamer DNA	Antisense ONT to P-gp	Direct, covalent	218	CCRF-CEM cell	ALL	in vitro	[45]
Sgc8, DNA	DNA nanotrains	Gold, DOX, DNR, and EPI	Direct, covalent	No data	PTK7	ALL	in vitro + in vivo	[46]
No name, DNA	Dextran-ferric oxide	None (HTT)	PDPH linker	≈70	HER2	SK-BR3 cells	in vitro	[47]
AS1411, DNA	PLGA-PEG	Vinorelbine	Direct, covalent	<200	Nucleolin	MDA-MB-231 cells	in vitro	[48]
No name, DNA	Liposome	TSP	Avidin-biotin interaction	No data	PDGFR	Breast cancer cells	in vitro	[49]
AS1411, DNA	PEGylated liposome	Anti-BRAF siRNA	Via PEG linker	≈150	Nucleolin	A375 tumor xenograft	in vivo	[50]
No name, RNA	PLGA-lecithin-PEG	Curcumin	Direct, covalent	90 ± 1.9	EpCAM	HT29 cells	in vitro	[51]
AS1411, DNA	pPEGMA-PCL-pPEGMA	Doxorubicin	Direct, covalent	≈140	Nucleolin	MCF-7 and PANC-1 cells	in vitro	[52]
AS1411, DNA	Gold nanoparticle	Doxorubicin or AZD8055	Dithiolane linker	No data	Nucleolin	MCF-7, el202 and OMM1.3	in vitro	[53]
A10, RNA and DUP-1	PEG-gold nanostar	None (PTT)	Direct, di- sulfide bonds	61.90 ±1.61	Prostate cell	Prostate cancer	in vitro	[54]
No name, DNA	Chitosan	SN38	Direct, covalent	≈200	Mucin-1	Colon cancer HT-29 cells	in vitro	[55]
AS1411, DNA	Gold nanoparticle	Doxorubicin and TMPyP4	Tethered by 21 bp DNA	38.7 ± 1.4	Nucleolin	HeLa and MCF-7R cells	in vitro	[15]
No name, RNA	Liposome	Doxorubicin	Tethered by linker DNA	90–100	PSMA	LNCaP cells	in vitro + in vivo	[56]
No name, DNA	Gold nanoparticle	Protein	With a His-tag	83.0 ± 1.3	His or GST	HeLa and A431 cells	in vitro + in vivo	[57]
A10–3.2, RNA	PEG-PAMAM	MicroRNA	Direct, covalent	177 ± 17.5	PSMA	Prostate cancer	in vitro + in vivo	[58]
A10–3.2, RNA	Atelocollagen	MicroRNA	Direct, covalent	221 ± 6.9	PSMA	Prostate cancer	in vivo	[59]
AS1411, DNA	PF127-β-CD-PEG-PLA	Doxorubicin	Covalent, to PF127	≈39.15	Nucleolin	MCF-7 cells	in vitro + in vivo	[60]
No name, RNA	PLGA	Nutlin-3a	Direct, covalent	292 ± 10	EpCAM	ZR751, MCF-7, SKOV3	in vivo	[61]
No name, RNA	PLGA-PEG	Doxorubicin	Direct, amide linking	136 ± 0.21	EpCAM	Non-small cell lung cancer	in vitro + in vivo	[62]
No name, RNA	PEI	EpCAM siRNA	Electrostatic interaction	198 ± 14.2	EpCAM	MCF-7 and WERI-Rb1 cells	in vivo	[63]
HB5, DNA	Mesoporous silica-carbon	Doxorubicin	thiol-amine link to PEG	≈140	HER2	SK-BR-3 cells	in vivo	[64]
No name, DNA	Au–GO	None (PTT)	Direct, Au–S bond	No data	Mucin-1	MCF-7 cells	in vivo	[65]
sgc8c, DNA	Gold nanorod	Hyperthermia therapy	Direct, Au–S bond	No data	CCRF-CEM cell	ALL	in vitro	[66] *
No name, RNA	PEG-PLGA	Doxorubicin	Direct, covalent	136 ± 0.21	EpCAM	MCF-7 cells	in vitro	[67]
AS1411, DNA	MOF shell, UCNP core	Doxorubicin	Direct, covalent	≈140	Nucleolin	MCF-7 and 293 cells	in vitro	[68]
No name, RNA	GPN	Gefitinib	Direct, covalent	No data	Ets1	H1975 cells	in vitro + in vivo	[69]
No name, DNA	Hyaluronan/Chitosan	5-fluorouracil	Direct, covalent	181	Mucin-1	Colorectal cancer	in vitro	[70]
Cy5.5-AS1411	GO and MSN	Doxorubicin	Non-covalent	No data	Nucleolin	MCF-7 cells	in vitro	[71]
A15, RNA	PLGA-PEG-COOH	Salinomycin	Direct, covalent	159.8	CD133	Osteosarcoma CSCs	in vitro + in vivo	[72]
S2.2, DNA	Graphene oxide-gold	Doxorubicin	Thiol–Au bonds	No data	Mucin-1	A549 and MCF-7 cells	in vitro	[73]
A15, CL4; RNA	PLGA	Salinomycin	Direct, covalent	139.7, 141.9	CD133, EGFR	Hepatocellular carcinoma	in vitro + in vivo	[74]
S2.2, DNA	ZnO nanoparticle	Doxorubicin	APTES linkage	5–10	Mucin-1	MCF-7 cells	in vitro	[75]
SRZ1, DNA	DOTAP:DOPE liposome	Doxorubicin	No data	≈100	4T1 cells	4T1 cells	in vitro + in vivo	[76]
AS1411, DNA	Tocopheryl PEG-PβAE	Docetaxel	No data	116.3 ± 12.4	Nucleolin	SKOV3 ovarian cancer cells	in vitro	[77]
No name, DNA	Chitosan and HA	SN38	Direct, covalent	129 ± 3.2	Mucin-1	HT29 cells	in vitro	[78]
S6, DNA	Dendrimer	MicroRNA	Direct, covalent	100–200	A549 cells	NSCLC cells	in vitro	[79]
AS1411, DNA	PLL-alkyl-PEI	shRNA	electrostatic coupling	168–183	Nucleolin	A549 cells	in vitro	[80]
AS1411, DNA	GQD-FMSN	Doxorubicin	Direct, amide bond	72.5	Nucleolin	HeLa cells	in vitro	[81]
KW16–13, DNA	PEG-gold nanorod	None (PTT)	Direct, covalent	No data	MCF10CA1h cell	Human breast duct carcinoma	in vitro	[82]
No name, DNA	Au-SPION	Gold for PTT	Thiol–Au interaction	≈39	Mucin-1	Colon cancer	in vitro	[83]
MA3	Iron	None (HTT)	Streptavidin-biotin, direct	≈296	Mucin-1	MCF-7 cells	in vitro	[84]
No name, RNA	Albumin	Cisplatin	Direct, amide bond	≈40	EGFR	Hela cell line	in vitro + in vivo	[85]
No name, DNA	Human IgG	miR29b	Indirect, C12 spacer	595.9 ±43.1	Mucin-1	A549 cells	in vitro	[86]
sgc8c and AS1411	Gold	Daunorubicin	Direct, covalent	No data	ALL and nucleolin	Molt-4 cells	in vitro	[87]
No name, RNA	Gold	Antisense ONT	Spacer, covalent	<50	CD33, CD34	AML-M2	in vitro	[88]
No name, DNA	Mesoporous silica	Doxorubicin	Direct, covalent	181 ± 6	EpCAM	SW620 colon cancer cells	in vitro	[89]
TSA14, RNA	PEGylated-liposome	Doxorubicin	Direct, covalent	118 ± 2.2	TUBO cells	Breast cancer	in vitro + in vivo	[90]
DNA-RNA hybrid	SPION	Doxorubicin	DNA linker, streptavidin-biotin	No data	PSMA	Prostate cancer	in vitro	[91]
AS1411, DNA	GC-rich dsDNA	Doxorubicin	Direct, covalent	6.1 ± 0.7; 7.4 ± 0.4	Nucleolin	Drug-resistant MCF-7 cells	in vitro	[92]
AS1411, DNA	PEG-PLGA	Gemcitabine	Direct, covalent	128 ± 5.23	Nucleolin	A549 cells	in vitro	[93]
AS1411, DNA	HPAEG	Doxorubicin	Direct, covalent	93.7	Nucleolin	MCF-7 and L929 cells	in vitro	[94]
No name, DNA	DNA dendrimer	Epirubicin	No data	36.4	MUC1, AS1411	MCF-7 and C26 cells	in vitro + in vivo	[95]
A10, RNA	PLGA	Triplex forming oligonucleotide	Direct, covalent	No data	PSMA	LNCaP cells	in vitro	[96]
No name, RNA	PLGA-PEG	Docetaxel	Direct, covalent	93.6	PSMA	LNCaP cells	in vitro + in vivo	[97]
AS1411, DNA	M-PLGA–TPGS	Docetaxel	Direct, covalent	130.1 ± 2.9	Nucleolin	HeLa cells	in vitro + in vivo	[98]
Endo28, DNA	3WJ-RNA	Doxorubicin	Direct, covalent	8.1 ± 1.5	Annexin A2	Ovarian cancer	in vitro + in vivo	[99]
No name, DNA	HAS-CS	Paclitaxel	Acrylate spacer	170 ± 4	Mucin-1	MCF-7 and T47D cells	in vitro	[100]
AS1411, DNA	PEG-PAMAM dendrimer	5-fluorouracil	Covalent, to PEG	No data	Nucleolin	Gastric cancer	in vitro	[101]
Two, DNA	DGL-PEG	Doxorubicin ATP-aptamer	Covalent, to PEG	≈38	Nucleolin, Cyt c	Nucleolin^+^ HeLa cells	in vitro + in vivo	[102]
No name, DNA	Iron oxide	None (HTT)	No data	No data	FGFR1	Human osteosarcoma	in vitro	[103]
A10, RNA	Liposome	CRISPR-Cas9 plasmid	Covalent, to DSPE-PEG	≈150	PSMA	Prostate cancer	in vitro + in vivo	[104]
AS1411, DNA	PEG-PAMAM dendrimer	Camptothecin	Covalent, to PEG	≈18	Nucleolin	HT29 and C26 cells	in vitro + in vivo	[105]
A6, DNA	Lipid-polymer liposome	siRNA	Direct, covalent	270 ± 10; 237 ± 12	HER2	SKBR-3 and 4T1-R cells	in vitro	[106]
No name, DNA	Chitosan- liposome	Erlotinib	Direct, covalent	179.4 ± 1.16	EGFR	EGFR-mutated cancer cells	in vitro	[107]
AS42, DNA	Gold	None (PTT)	No data	≈37	Ehrlich’s ACC	Ehrlich carcinoma	in vivo	[108]
No name, DNA	MCS nanogel	Doxorubicin	Direct, covalent	15–25	LNCaP cell	Prostate cancer	in vitro	[109]
AS1411 + S2.2, DNA	Gold-coated liposome	Docetaxel	through S-Au bond	≈200	Mucin-1, Nucleolin	MCF-7 cells	in vitro + in vivo	[110]
5TR1, DNA	PLGA-chitosan	Epirubicin	Electrostatic coupling	≈222.7	Mucin-1	MCF-7 and C26 cells	in vitro + in vivo	[111]
AS1411, DNA	Alkyl PAMAM dendrimer	Bcl-xL shRNA	Covalent and non-covalent	148–230	Nucleolin	A549 cells	in vitro	[112]
Gint4.T	PLGA-PEG-COOH	PI3K-mTOR inhibitor	Direct, covalent	52 ± 1	PGFRβ	Glioblastoma U87MG cells	in vitro + in vivo	[113]
No name, DNA	Mesoporous silica	Epirubicin	Via disulfide bonding	258.5 ± 20.1	Mucin-1	MCF-7 cells	in vitro	[114]
No name, DNA	Aminopropyl MSN	Safranin O	electrostatic + H-bonding	≈407	Mucin-1	MDA-MB-231 cells	in vitro	[115]
No name, DNA	Chitosan- liposome	PFOB and Erlotinib	Direct, covalent	≈180	EGFR	NSCLC cell lines	in vitro + in vivo	[116]
No name, DNA	Au-Fe_3_O_4_	None	Electrostatic absorption	46 ± 3	VEGF	SKOV-3 ovarian cancer cells	in vitro	[117]
No name, DNA	MPC-PAA/PEI	Doxorubicin	Anchoring via EHH	No data	Mucin-1	A549 and MCF-7 cells	in vitro	[118]
A15, RNA	PLGA	Propranolol	Direct, covalent	143.7± 24.6	CD133	Hemangioma	in vitro + in vivo	[119]
AIR-3A, RNA	PEG-coated gold NP	None	Thiol–gold bonds	2, 7, 36	IL-6R	IL-6R-carrying cells	in vitro	[120]
No name, DNA	PDA/PEG- coated MSN	DM1	Direct, covalent	203.75 ±2.37	EpCAM	Colorectal cancer	in vitro + in vivo	[121]
AS-14, DNA	Gold-coated magnetic NP	None, using magnetic field	Thiolated ONT primer	50 (GMNP)	Fibronectin protein	Ehrlich carcinoma	in vivo	[122]
AS1411, DNA	Chitosan-ss-PEEUA	TLR4-siRNA, Doxorubicin	Direct, covalent	124.6 ± 1.068	Nucleolin	A549 cells	in vitro + in vivo	[123]
FKN-S2, DNA	PEG-aptamer micelle	None or Aptamer	ssDNA-amphiphile	No data	Fractalkine	Colon adeno-carcinoma	in vitro + in vivo	[124]
No name, DNA	Ursolic acid, Doxorubicin	Ursolic acid, Doxorubicin	Electrostatic interactions	≈108.9	HER2	HER2-carrying cells	in vitro + in vivo	[125]
No name, DNA	PEG-SPION	Doxorubicin	Direct, covalent	5–64	Mucin-1	MCF-7 cells	in vitro	[126]
Two, DNA	NMOF	Doxorubicin	Hybridization	≈130	Nucleolin, VEGF	MDA-MB-231	in vitro	[127]
5TR1, DNA	PEI-PEG and Na_2_SeO_3_	Epirubicin and an aptamer	Covalent, to PEG	No data	Mucin-1	MCF-7 and C26 cells	in vitro + in vivo	[128]
No name, DNA	Liposome	Doxorubicin	Amino- carboxyl	170 ± 25	HER3	MCF-7 breast cancer cells	in vitro + in vivo	[129]
No name, DNA	DNA nano-ring	Doxorubicin	Incorporated in DNA ring	≈29 (DNA ring)	Mucin-1	MCF-7 breast cancer cells	in vitro	[130]
A10–3.2, RNA	Cationic nanobubble	FoxM1 siRNA	Direct, covalent	479.83 ± 24.50	PSMA	LNCaP cells	in vitro + in vivo	[131]
No name, DNA	DNA micelle	Doxorubicin, KLA peptide	No data	371	Mucin-1	MCF-7 cells	in vitro + in vivo	[132]
No name, DNA	Lipid-polymer	Salinomycin	Thiolated, direct	96.3 ± 9.8	CD20	Melanoma stem cells	in vitro + in vivo	[133]
No name, RNA	Polymer-lipid	Salinomycin	Thiolated, direct	95	EGFR	Osteosarcoma CSCs	in vitro	[134]
trCLN3, DNA	Lipidated GC-rich DNA hairpin	Doxorubicin, 2′,6′-dimethyl azobenzene	Lipid-mediated self-assembly	21.2 ± 1.5	cMet	cMet-expressing H1838 cells	in vitro	[135]
TLS1c, DNA	Liposome	Cabazitaxel	Avidin-biotin interaction	90.10 ± 2.71	MEAR cells	Hepatoma	in vitro + in vivo	[136]
No name, DNA	PBABT	Docetaxel	Direct, covalent	274.7 ± 46.1	HER2	Epithelixal ovarian cancer	in vitro + in vivo	[137]
No name, DNA	BSA-PEG-Fe^3+^	Mn, Doxorubicin	GAG-linker, base-match	No data	Glut-1	HepG-2 cells	in vitro + in vivo	[138]
AS1411	TD-PEC- chitosan	miR-145	Electrostatic bonds with chitosan	40–270	Nucleolin	MCF-7 cells	in vitro + in vivo	[139]
No name, DNA	DNA	ALK-siRNA, Doxorubicin	Direct, covalent	59	CD30	ALCL	in vitro + in vivo	[140]
No name, DNA	Human IgG	MicroRNA	Direct, covalent	595	Mucin-1	Non-small cell lung cancer	in vitro + in vivo	[141]
S15, DNA	Quantum dots	None	Direct, covalent	No data	NSCLC	A549 cells	in vitro	[142]
A15, CL4; RNA	Lipid-polymer	Salinomycin	Direct, covalent	110.2 ± 12.1	CD133, EGFR	Osteosarcoma cells and CSCs	in vitro + in vivo	[143]
No name, DNA	PEG-Au- PAMAM	Curcumin	Covalent, C6 linker	5.23 ± 4.12	Mucin-1	HT29 and C26 cells	in vitro + in vivo	[144]
No name, RNA	Liposome	Docetaxel	Covalent, to DSPE-PEG	116.5 ± 9.3	CD133	A549 cells	in vitro + in vivo	[145]
5TR1, DNA	PEGylated liposome	Doxorubicin	No data	120 ± 1.8	Mucin1	C26 cells	in vitro + in vivo	[146]
AS1411, DNA	Bovine serum albumin	Doxorubicin	Direct, amidation	163 ± 2.5	Nucleolin	MCF-7 cells	in vitro	[147]
No name, DNA	Copper oxide	mRNA 29b	Direct, amide linking	≈40	Mucin 1	A549 cells	in vitro	[148]
Sgc8c, DNA	Fe_3_O_4_-carbon	Doxorubicin	Direct, covalent	No data	No data	A549 cells	in vitro + in vivo	[149]
A9, RNA	Gold	None (PTT)	No data	≈70	PSMA	LNCaP cells	in vitro	[150]
No name, DNA	Gold nanoshell	None (PTT)	Direct, thiol–Au bonds	No data	Mucin 1	A549, MCF-7 3D cell culture	in vitro	[151]
C10.36, DNA	PAM (peptide + DNA ONT)	Peptide	Base pairing	110 ± 30	HBLL	B-cell leukemia cells	in vitro	[152]
No name, RNA	LP-DNA	SATB1 siRNA	Thiolated, direct	161.2 ± 11.3	EGFR	Choriocarcinoma	in vivo	[153]
AS1411, DNA	PEGylated PLGA	anti-miR-21, cisplatin (CIS)	Direct, covalent	142.4 ± 5.9 106.6 ± 5.9	Nucleolin	CIS-resistant A2780 cells	in vitro	[154]
No name, RNA	Lipid-PLGA	All-trans retinoic acid	Thiolated, direct	129.9	CD133	Lung cancer initiating cells	in vitro	[155]
S15, DNA	PEG-PCL	Paclitaxel	Direct, amide linking	≈15	NSCLC	A549 cells	in vitro	[156]
S2.2, DNA	Elastin-like polypeptide	Paclitaxel	Via gene A’ protein	No data	Mucin-1	MCF-7 cells	in vitro	[157]
5TR1, DNA	PβAE and PLGA	Epirubicin, antimir-21	Direct, covalent	210.4 ±10.14	Mucin-1	MCF-7 cells	in vitro + in vivo	[158]
No name, RNA	Lipid-polymer	All-trans retinoic acid	Thiolated, direct	129.9	CD133	Osteosarcoma initiating cells	in vitro	[159]
No name, DNA	Calcium carbonate	Epirubicin, and melittin	Avidin-biotin interaction	>300	Mucin-1	MCF-7 and C26 cells	in vitro + in vivo	[160]
ACE4	Diacetylene-PEG	None	31 G spacer, base pairing	≈13	Annexin A2	MCF-7 cells	in vitro	[161]
No name, DNA	Human IgG	Genistein and miRNA-29b	C12 spacer, covalent	598 ± 34.1	Mucin-1	A549 cell line	in vitro	[162]
No name, DNA	Lipid-quantum dot	siRNA	Direct, covalent	No data	EGFR	Triple-negative breast cancer	in vitro + in vivo	[163]
HB5, DNA	Human serum albumin	Curcumin	Direct, covalent	281.1 ± 11.1	HER2	SK-BR-3 cells	in vitro	[164]
AS1411, DNA	Magnetic SPION/MSN	Doxorubicin	Direct, covalent	89	Nucleolin	MCF-7 cells	in vitro	[165]
AS1411, DNA	Albumin-IONP/GNP	Doxorubicin	Direct, covalent	≈120	Nucleolin	MCF-7 and SKBR3 cells	in vitro	[166]
C2NP, DNA	PEG-PLGA	Doxorubicin	Direct, covalent	168.07 ± 2.72	CD30	Large cell lymphoma	in vitro	[167]
AS1411, DNA	Liposome	Paclitaxel and PLK1 siRNA	DSPE-PEG-MAL	121.27 ± 2.51	Nucleolin	MCF-7 cells	in vitro + in vivo	[168]
AS1411, DNA	Liposome	Aptamer- doxorubicin	Not Applicable	≈128.6	Nuclear nucleolin	MCF-7/Adr cells	in vitro	[169]
AS1411, DNA	PEGylated liposome	5-fluorouracil	Via PEG linker	190 ± 15	Nuclear nucleolin	Basal cell carcinoma	in vitro	[170]
HApt, DNA	β-CD-capped MSN	Doxorubicin	Thiolated to β-CD	218.2 ±6.1	HER2	HER2-positive cells	in vitro	[16]
AS1411, DNA	SPION	Daunomycin, TMPyP	Amide bond, direct	15–20	Nucleolin	A549 and C26 cells	in vitro	[171]
S1.5, DNA	PEGylated PLGA	Docetaxel	Carbodiimide coupling	142.7± 12.3	HPA	TNBC cells	in vitro + in vivo	[172]
No name, DNA	Mesoporous MnO_2_	HMME	Direct, covalent	≈200	Mucin 1	MCF-7 cells	in vitro + in vivo	[173]
AS1411, DNA	PLGA, PVP	Doxorubicin	Direct, covalent	≈87.168	Nucleolin	A549 cells	in vitro + in vivo	[174]
No name, DNA	DNA hydrogel	CpG ONT and Doxorubicin	Covalent, to CpG ONT	50.1 ± 2.82	Mucin-1	MCF-7 cells	in vitro	[175]
AS1411, DNA; (HA)	Micro-emulsion	Shikonin and docetaxel	Direct, thiolated	≈30	Nucleolin; (CD44)	Glioma	in vitro, model	[176]
No name, DNA	Cationic liposome	miR-139–5p	Direct, covalent	150.3 ±8.8	EpCAM	Colorectal Cancer	in vitro + in vivo	[177]
Sgc8, DNA	MSN	Doxorubicin	Direct, covalent	103.24	PTK7	CCRF-CEM cells	in vitro	[178]
GMT8, Gint4.T; DNA	DNA	Paclitaxel	Direct, covalent	17.78	U87MG cell, PDGFRβ	Glioblastoma	in vitro	[179]
AS1411, DNA	Derived from erythrocytes	Doxorubicin, siRNA	Covalent to cholesterol via 6-A bases	≈100	Nucleolin	MDR MCF-7 cells	in vitro	[180]
TA6, DNA	DNA nanotrain	AKT inhibitor, Doxorubicin	Direct, covalent	No data	CD44	Breast cancer stem cells	in vitro + in vivo	[181]
A15, RNA	Liposome	Curcumin	Direct, thiol-maleimide	86.6 ± 4.5	CD133	DU145 cells	in vitro + in vivo	[182]
AS1411, DNA	Silver-PEG	None (irradiation)	Amide bond to PEG	18.82 ± 2.1	Nucleolin	Glioma	in vitro + in vivo	[183]
U2, DNA	Gold	None	Direct, Au-S bond	≈60.23	EGFR	Glioblastoma	in vitro + in vivo	[184]
M49, DNA	PEGylated liposome	Doxorubicin	Covalent, to PEG	No data	CD200R1	4THM breast carcinoma	in vivo	[185]
TC01, Sgc4f, and Sgc8; DNA	DNA ONT	Doxorubicin	DNA ONT hybridization	No data	Multiple cancers and PTK7	CCRF-CEM cells	in vitro + in vivo	[186]
No name, DNA	DNA origami	Antisense ONT, doxorubicin	Extended sequences	4.17 ± 0.12 (height)	Mucin-1	HeLa/ADR cells	in vitro	[187]
LZH5B, DNA	DNA nanotrain	Doxorubicin	Hybridization	No data	HepG2 cell	HepG2 cell line	in vitro	[188]
No name, DNA	SPION@SiO_2_	Doxorubicin	Direct, covalent	5–27	Mucin-1	MCF-7 cells	in vitro	[189]
AS1411, DNA	Upconversion nanoparticle	Protoporphyrin IX	Direct, covalent	120 ± 4	Nucleolin	HeLa and A549 cells	in vitro	[190]
AS-14, AS-42; DNA	SPMFN	Doxorubicin	Glycosidic linkages	No data	FN, HSP71	Ehrlich carcinoma cells	in vitro + in vivo	[191]
AS1411, DNA	Gold	Anti-miR-155	PolyA linker sequence	≈30	Nucleolin	MCF-7 cells	in vitro	[192]
L5, etc., DNA	PLGA	Docetaxel	Direct, covalent	156.9 ± 42.97	Not clear yet	HepG2 and Huh-7 cells	in vitro + in vivo	[193]
L5, DNA	PLGA	Docetaxel	Direct, covalent	211.9–236.1	TAG-72	HepG2 and Huh-7 cells	in vitro	[194]
LXL, DNA	RNA hydrogel	siRNA and miRNA	No data	≈200	MDA-MB-231 cell	Triple-negative breast cancer	in vitro + in vivo	[195]
AS1411, DNA	CaCO_3_ and protamine	CRISPR-Cas9 plasmid	Covalent, to HA	230–320	Nucleolin	H1299 cells	in vitro	[196]
No name, RNA	Hollow gold nanosphere	Doxorubicin	Thiolated	≈42 (25–55)	CD30	Karpas 299 cells	in vitro	[197]
C2NP, DNA	DNA nanotube	Doxorubicin	By extending staples	140 × 14 (L × W)	CD30	K299 cells	in vitro	[198]
No name, DNA	ssDNA-ELP	Docetaxel	Covalent, to ELP	10–40	Mucin-1	MCF-7 cells	in vitro	[199]
No name, DNA	Magnetic nanosphere	Doxorubicin	Streptavidin-biotin	No data	EpCAM	MCF-7 cells (CTCs)	in vitro	[200]
AS1411, DNA	DNA nanotrains	DOX, EPI, and DAU	Base pairing	No data	Nucleolin	HeLa cells	in vitro	[201]
No name, RNA	Protamine	Doxorubicin, ALK-siRNA	Non-covalent	No data	CD30	ALCL	in vitro	[202]
AS1411, DNA	TiO_2_ nanofiber with BSA	None	Streptavidin-biotin	81.33 ± 25.70	AS1411, DNA	MCF-7 cells (CTCs)	in vitro	[203]
AS1411, DNA	Gold and liposome	Morin	Covalent, Au-S	No data	Nucleolin	SGC-7901 cells	in vitro + in vivo	[204]
AS1411, DNA	GO Nanosheet	Berberine derivative	NH_2_-(CH_2_)_6_ linker	30–50 × 2–3	Nucleolin	A549 cells	in vitro	[205]
AS1411, DNA	DNA Holliday junction	Doxorubicin	Phospho-diester bond	12.45 ± 2.16	Nucleolin	CT26 colon cancer cells	in vitro	[206]
Syl3c, DNA	PEGylated liposome	Doxorubicin	Covalent, to PEG	110 ± 5	EpCam	C26 Colon Carcinoma	in vitro + in vivo	[207]
No name, DNA	Ag-MOF-RBCm	PFK15	Inserted into RBCm	≈109	CD20	B-cell lymphoma	in vitro + in vivo	[208]
No name, DNA	PCL-MMA/MPEG-MASI	Doxorubicin	Covalent, to NHS group	≈124	EpCAM	HT29 cells	in vitro	[209]
AS1411, DNA	FO-loaded MOF-RBCm	Using PDT and CDT effects	Inserted via cholesterol	110–140	Nucleolin	KB cells	in vitro + in vivo	[210]
MAGE-A3, DNA	NIR PLN	Afatinib	By a disulfide bond	225	MAGE	NSCLC	in vitro + in vivo	[211]
A10-3.2, RNA	Lipid-polymer hybrid	Curcumin and Cabazitaxel	Covalent, to PEG	121.3 ± 4.2	PSMA	Prostate cancer	in vitro + in vivo	[212]
A6, DNA	DOTAP, Mal-PEG, cholesterol, PLGA	P-gp siRNA	Covalent, to Mal-PEG	No data	HER2	DOX-resistant 4T1 cells	in vitro	[213]
Wy5a, DNA	PLGA-PEG-COOH	Docetaxel	Amide bond with spacer	≈154.3	PC-3 cell	Prostate cancer	in vitro + in vivo	[214]

The aptamers in the table are listed in the order they appear in the literature. ⱡ Size of the nanoparticles after aptamer conjugation. For spherical nanoparticle, the number is the diameter of the particle; for nanotubes or nanosheets, the measurement uses a × symbol. ^#^ Direct conjugation means there is no bridge, spacer, or linker molecule/sequence between the aptamer and the nanoparticle. * The aptamer-conjugated gold nanorods were surface modified with BSA through electrostatic interactions.

**Table 2:** Aptamer-functionalized nanoparticles classified by nanomaterials and payloads.

Type of Nanoparticle		Payloads	Aptamers	Targets	Cancers	References
Polymer based nanoparticles	PLA-PEG	Rhodamine-labeled dextran	A10	PSMA	Prostate cancer,	[18]
	PLGA-PEG	Cisplatin, Docetaxel, Doxorubicin, Gemcitabine, Paclitaxel, Salinomycin, Vinorelbine, PI3K-mTOR inhibitor, anti-miR-21, and cisplatin,	A10, A15, AS1411, C2NP, EpCAM-Ap, Gint4.T, PSMA-Ap, S1.5, Wy5a	CD30, CD133, EpCAM, HPA, Nucleolin, PC-3 cell, PGFRβ, PSMA	Breast cancer, glioblastoma, glioma, large cell lymphoma, lung cancer, NSCLC, osteosarcoma, cisplatin-resistant ovarian cancer, prostate cancer, TNBC	[22,29,35,57,71,76,81,102,6,122,163,176,181,223]
	PLGA	Docetaxel, Paclitaxel, Nutlin-3a, Salinomycin, Triplex forming oligonucleotide, Propranolol	A10, A15, AS1411, L5, S2.2, EpCAM-Ap	PSMA, CD133, EGFR, MUC1, Nucleolin, TAG-72	Breast cancer, hepatocellular carcinoma, hemangioma, human glial cancer, prostate cancer	[34,42,83,105,128,203]
	PEG-PCL	Docetaxel	AS1411, GMT8, S15	Nucleolin, NSCLC, U87 cells	Glioblastoma, glioma, lung cancer	[43,45,165]
	H40-PLA-PEG	Doxorubicin	A10	PSMA	Prostate cancer	[42]
	pPEGMA-PCL-pPEGMA	Doxorubicin	AS1411	Nucleolin	Pancreatic carcinoma	[52]
	PEG-PAMAM	MicroRNA	A10–3.2	PSMA	Prostate cancer	[58]
	PF127-β-CD-PEG-PLA	Doxorubicin	AS1411	Nucleolin	Breast cancer	[60]
	PEI	EpCAM-siRNA	EpCAM-Ap	EpCAM	Breast cancer, retinoblastoma	[63]
	GPN	Gefitinib	Ets1-Ap	Ets1	NSCLC	[69]
	PLL-alkyl-PEI	shRNA	AS1411	Nucleolin	Lung cancer	[80]
	HPAEG	Doxorubicin	AS1411	Nucleolin	Breast cancer	[94]
	M-PLGA–TPGS	Docetaxel	AS1411	Nucleolin	Cervical cancer	[98]
	PBABT	Docetaxel	HER2-Ap	HER2	Ovarian cancer	[137]
	PβAE and PLGA	Epirubicin and antimir-21	5TR1	MUC1	Breast cancer	[158]
	PLGA, PVP	Doxorubicin	AS1411	Nucleolin	Lung cancer	[174]
	PCL-MMA/MPEG-MASI	Doxorubicin	EpCAM-Ap	EpCAM	Colorectal cancer	[209]
Lipid based nanoparticles	Liposome	Curcumin, Doxorubicin, Cabazitaxel, Cisplatin, CRISPR-Cas9 plasmid, Docetaxel, Doxorubicin, Paclitaxel, and PLK1 siRNA, TSP	A10, A15, AS1411, HER3-Ap, PSMA-Ap, TLS1c	CD133, HER3, MEAR cells, Nucleolin, PSMA, PDGFR	Breast cancer, DOX-resistant breast cancer, Hepatoma, lung cancer, prostate cancer,	[33,50,58,65,113,138,145,154,178,191]
	PEGylated-liposome	5-FU, Doxorubicin, Anti-BRAF siRNA	5TR1, AS1411, M49, Syl3c, TSA14,	CD200R1, EpCAM, Mucin1, Nucleolin, TUBO cells	Basal cell carcinoma, breast cancer, colon carcinoma, melanoma	[50,90,146,170,185,207]
	DOTAP:DOPE liposome	Doxorubicin	SRZ1	4T1 cells	Breast cancer	[76]
	Cationic liposome	miR-139–5p	EpCAM-Ap	EpCAM	Colorectal Cancer	[177]
Chitosan based nanoparticles	Chitosan	SN38	MUC1-Ap	MUC1	Colon cancer	[55]
	Chitosan and HA	SN38	MUC1-Ap	MUC1	Colorectal adenocarcinoma	[78]
	HAS-CS	Paclitaxel	MUC1-Ap	MUC1	Breast cancer	[100]
Dendrimer based nanoparticles	Dendrimer	MicroRNA	S6, sgc8c	A549 cell, CCRF-CEM	ALL, NSCLC	[23,79]
	ONT-PAMAM dendrimer	Doxorubicin	A9	PSMA	Prostate cancer	[29]
	PEG-PAMAM dendrimer	5-fluorouracil, Camptothecin	AS1411	Nucleolin	Colorectal cancer, Gastric cancer	[101,105]
	DGL-PEG	Doxorubicin, ATP-aptamer	AS1411, Cyt c-Ap	Nucleolin, Cyt c	Cervical cancer	[102]
	Alkyl PAMAM dendrimer	Bcl-xL shRNA	AS1411	Nucleolin	Lung cancer	[112]
Hydrogel based nanoparticles	MCS nanogel	Doxorubicin	LNCaP-Ap	LNCaP cell	Prostate cancer	[109]
	DNA Hydrogel	CpG ONT and Doxorubicin	MUC1-Ap	MUC1	Breast cancer	[175]
	RNA Hydrogel	siRNA and miRNA	LXL	MDA-MB-231 cell	Triple-negative breast cancer	[195]
Nucleic acid based nanoparticles	DNA icosahedra	Doxorubicin	MUC1-Ap	MUC1	Breast cancer	[27]
	Aptamer DNA	Antisense ONT against P-gp	sgc8c	CCRF-CEM cell	ALL	[45]
	GC-rich dsDNA	Doxorubicin	AS1411	Nucleolin	Drug-resistant breast cancer	[92]
	DNA dendrimer	Epirubicin	MUC1-Ap, AS1411-Ap	MUC1, AS1411	Breast and colon cancers	[95]
	3WJ-RNA	Doxorubicin	Endo28	Annexin A2	Ovarian cancer	[99]
	DNA nano-ring	Doxorubicin	MUC1-Ap	MUC1	Breast cancer	[130]
	Lipidated GC-rich DNA hairpin	Doxorubicin and 2′,6′-dimethyl-azobenzene	trCLN3	cMet	cMet-expressing lung cancer	[135]
	DNA	ALK-siRNA, Doxorubicin, Paclitaxel	CD30-Ap, Gint4.T, GMT8, Sgc4f, Sgc8, TC01	cancer cells, CD30, PDGFRβ, PTK7, U87MG cell	ALCL, ALL, Glioblastoma	[140,179,186]
	DNA origami	Antisense ONT, doxorubicin	MUC1-Ap	MUC1	MDR cervical cancer	[187]
	DNA nanotube	Doxorubicin	C2NP	CD30	Human anaplastic large cell lymphoma	[198]
	DNA nanotrain	AKT inhibitor, DAU, DOX, DNR, EPI, Gold	AS1411, LZH5B, Sgc8, TA6	CD44, HepG2 cell, nucleolin, PTK7	ALL, Breast cancer stem cell, cervical cancer, liver cancer	[46,181,188,201]
	DNA Holliday junction	Doxorubicin	AS1411	Nucleolin	Colon cancer	[206]
Protein/peptide based nanoparticles	Albumin	Cisplatin, Curcumin, Doxorubicin	AS1411, EGFR-Ap, HB5	EGFR, HER2, nucleolin	Breast cancer, cervical cancer	[85,147,164]
	Human IgG	Genistein, miRNA-29b	MUC1-Ap	MUC1	NSCLC	[86,141,162]
	Elastin-like polypeptide	Paclitaxel	S2.2	MUC1	Breast cancer	[157]
	Human serum albumin					
	Protamine	Doxorubicin, ALK-siRNA	CD30-Ap	CD30	Lymphoma	[202]
Polymer and lipid hybrids	PLGA-lecithin-PEG	Paclitaxel, Curcumin	AS1411, EpCAM	Nucleolin	Breast cancer, colorectal adenocarcinoma	[32,51]
	PLGA-lipid-PEG	Docetaxel	XEO2mini	PC3 cells	Prostate cancer	[31]
	Lipid-polymer liposome	siRNA	A6	HER2	Breast cancer	[106]
	Polymer-lipid	All-trans retinoic acid, Curcumin and Cabazitaxel, Salinomycin	A10–3.2, A15, CD20-Ap, CD133-Ap, CL4, EGFR-Ap	CD20, CD133, EGFR, PSMA	Melanoma, osteosarcoma, prostate cancer	[133,134,143,159,212]
	Lipid-PLGA	All-trans retinoic acid	CD133-Ap	CD133	Lung cancer	[155]
	DOTAP, PLGA, cholesterol, Mal-PEG	P-gp siRNA	A6	HER2	DOX-resistant breast cancer	[213]
Polymer and chitosan hybrids	PLGA-chitosan	Epirubicin	5TR1	MUC1	Breast cancer, colon carcinoma	[111]
	Chitosan-ss-PEEUA	TLR4-siRNA, Doxorubicin	AS1411	Nucleolin	Lung cancer	[123]
Chitosan and lipid hybrids	Chitosan-liposome	Erlotinib	EGFR-Ap	EGFR	EGFR-mutated cancer cells	[107]
	Chitosan-liposome	PFOB and Erlotinib	EGFR-Ap	EGFR	NSCLC	[116]
Nucleic acid and peptide hybrids	KLA-DNA micelle	Doxorubicin+KLA	MUC1-Ap	MUC1	Breast cancer	[132]
	PAM (peptide +DNA ON)	Peptide	C10.36	HBLL	B-cell leukemia	[152]
	ssDNA-ELP	Docetaxel	MUC1-Ap	MUC1	Breast cancer	[199]
Other organic nanoparticles	Atelocollagen	MicroRNA	A10–3.2	PSMA	Prostate cancer	[59]
	Tocopheryl PEG-PβAE	Docetaxel	AS1411	Nucleolin	Ovarian cancer	[77]
	PEG-aptamer micelle	None or Aptamer	FKN-S2	Fractalkine	Colon adeno-carcinoma	[124]
	Ursolic acid	Doxorubicin	HER2-Ap	HER2	HER2-carrying cells	[125]
	TD-PEC-chitosan	miR-145	AS1411	Nucleolin	Breast cancer	[139]
	LP-DNA	SATB1 siRNA	EGFR-Ap	EGFR	Choriocarcinoma	[153]
	Diacetylene-PEG	None	ACE4	Annexin A2	Breast cancer	[161]
Inorganic nanoparticles	Au-Ag	Photothermal therapy	sgc8c	CCRF-CEM cell	ALL	[21]
	Gold	Anti-miR-155, Antisense ONT, Daunorubicin, Doxorubicin, TMPyP4, PTT	A9, AIR-3A, AS1411, As42, CD30-Ap, CD33/CD34-Ap, KW16–13, MUC1-Ap, sgc8c, U2	CCRF-CEM, CD30, CD33/CD34, EGFR, Ehrlich’s ACC, IL-6R, MCF10CA1h, MUC1, nucleolin, PSMA	ALL, AML, breast cancer, cervical cancer, Ehrlich carcinoma, glioblastoma, human breast duct carcinoma, lymphoma, lung cancer, prostate cancer	[15,44,53,54,66,82,87,88,108,120,150,151,184,192,197]
	Mesoporous silica	Doxorubicin, Epirubicin, Fluorescein, gold nanorods	AS1411, Sgc8, EpCAM-Ap, MUC1-Ap	Nucleolin, PTK7, EpCAM, MUC1	ALL, breast cancer, human T cell leukemia, colon cancer	[35,38,39,40,89,114,178]
	Mesoporous silica–carbon	Doxorubicin	HB5	HER2	Breast cancer	[64]
	Graphene oxide-gold	Doxorubicin, None (PTT)	S2.2, MUC1-Ap	MUC1	Breast cancer, lung cancer	[65,73]
	Graphene oxide-MSN	Doxorubicin	Cy5.5-AS1411	Nucleolin	Breast cancer	[71]
	ZnO	Doxorubicin	S2.2	MUC1	Breast cancer	[75]
	GQD-FMSN	Doxorubicin	AS1411	Nucleolin	Cervical cancer	[81]
	Iron	None (HTT)	MA3	MUC1	Breast cancer	[84]
	Au-Fe_3_O_4_	None	VEGF-Ap	VEGF	Ovarian cancer	[117]
	Copper oxide	mRNA 29b	MUC1-Ap	MUC1	Lung cancer	[148]
	Calcium carbonate	Epirubicin, and melittin	MUC1-Ap	MUC1	Breast cancer	[160]
	Mesoporous MnO_2_	HMME	MUC1-Ap	MUC1	Breast cancer	[173]
	Silver-PEG	Irradiation	AS1411	Nucleolin	Glioma	[183]
	Graphene oxide sheets	Berberine derivative	AS1411	Nucleolin	Lung cancer	[205]
Quantum dot based nanoparticles	Quantum dots	None	S15	NSCLC	Lung cancer	[142]
	QD-PMAT-PEI	siRNA	PSMA-Ap	PSMA	Prostate cancer	[30]
	Lipid-quantum dot	siRNA	EGFR-Ap	EGFR	Triple-negative breast cancer	[163]
Magnetic nanoparticles	SPION	Epirubicin, Doxorubicin, Daunomycin and TMPyP	5TR1, A9, A10, AS1411, DNA-RNA hybrid	MUC1, Nucleolin, PSMA	Colon cancer, breast cancer, lung cancer, prostate cancer	[22,28,43,91,171]
	Dextran-ferric oxide	None	HER2-Ap	HER2	Human adenocarcinoma	[47]
	Au-SPION	None	MUC1-Ap	MUC1	Colon cancer	[83]
	Iron oxide	None (HTT)	FGFR1-Ap	FGFR1	Human osteosarcoma	[103]
	Gold-coated magnetic NP	None	AS-14	Fibronectin protein	Ehrlich carcinoma	[122]
	PEG-SPION	Doxorubicin	MUC1-Ap	MUC1	Breast cancer	[126]
	Fe3O4-carbon	Doxorubicin	Sgc8c-Ap	Sgc8c	Lung cancer	[149]
	Magnetic SPION/MSN	Doxorubicin	AS1411	Nucleolin	Breast cancer	[165]
	SPMFN	Doxorubicin	AS-14 and AS-42	FN and HSP71	Ehrlich carcinoma	[191]
	SPION@SiO_2_	Doxorubicin	MUC1-Ap	MUC1	Breast cancer	[189]
	Magnetic nanosphere	Doxorubicin	EpCAM-Ap	EpCAM	Breast cancer	[200]
Other inorganic nanoparticles	Gd:SrHap	Doxorubicin	AS1411	Nucleolin	Breast cancer	[37]
Organic and inorganic hybrids	MOF-UCNP	Doxorubicin	AS1411	Nucleolin	Breast cancer	[68]
	Gold-liposome	Docetaxel, Morin	AS1411, S2.2	Nucleolin, MUC1	Breast cancer, gastric cancer	[119,213]
	Aminopropyl MSN	Safranin O	MUC1-Ap	MUC1	Breast cancer	[115]
	MPC-PAA/PEI	Doxorubicin	MUC1-Ap	MUC1	Breast cancer, lung cancer	[118]
	PDA/PEG- coated MSN	DM1	EpCAM-Ap	EpCAM	Colorectal cancer	[121]
	NMOF	Doxorubicin	AS1411, VEGF-Ap	Nucleolin, VEGF	Breast cancer	[127]
	PEI-PEG and Na_2_SeO_3_	Epirubicin and an aptamer	5TR1	MUC1	Breast cancer	[128]
	BSA-PEG-Fe^3+^	Mn, Doxorubicin	Glut-1-Ap	Glut-1	Liver cancer	[138]
	PEG-Au- PAMAM	Curcumin	MUC1-Ap	MUC1	Colon adenocarcinoma	[144]
	Albumin-IONP/GNP	Doxorubicin	AS1411	Nucleolin	Breast cancer	[166]
	β-CD-capped MSN	Doxorubicin	HApt	HER2	HER2-positive cells	[16]
	CaCO_3_ and protamine	CRISPR-Cas9 plasmid	AS1411	Nucleolin	NSCLC	[196]
	TiO_2_ nanofiber with BSA	None	AS1411	Nucleolin	Breast cancer CTCs	[203]
Others	Cationic nanobubble	FoxM1 siRNA	A10–3.2	PSMA	Prostate cancer	[131]
	Micro-emulsion	Shikonin and docetaxel	AS1411 and HA	Nucleolin and CD44	Glioma	[176]
	RBC membrane	Doxorubicin, siRNA	AS1411	Nucleolin	MDR breast cancer	[180]
	Upconversion nanoparticle	Protoporphyrin IX	AS1411	Nucleolin	Cervical cancer, lung cancer	[190]
	Ag-MOF-RBCm	Doxorubicin	CD20-Ap	CD20	B-cell lymphoma	[208]
	FO-loaded MOF-RBCm	Using PDT and CDT effects	AS1411	Nucleolin	KB Cell Line	[210]
	NIR PLN	Afatinib	MAGE-A3	MAGE	NSCLC	[211]

For aptamers that do not have a name, “target-Ap” is used to represent the aptamer; for example, EpCAM-Ap represents the aptamer that targets EpCAM.

## Abbreviations

3WJ-RNA	a highly stable three-way junction (3WJ) motif from phi29 packaging RNA
5-FU	5-fluorouracil
ALCL	anaplastic large cell lymphoma
ALK	anaplastic lymphoma kinase
ALL	acute lymphoblastic leukemia, also known as T-cell acute lymphoblastic leukemia
AML-M2	acute myeloid leukemia subtype 2
APTES	(3-aminopropyl) triethoxysilane
BSA	bovine serum albumin
cMet	hepatocyte growth factor receptor
COOH	(terminal) carboxylic acid group
CSC	cancer stem cell
CTC	circulating tumor cell
CUR-NP	curcumin-loaded lipid-polymer-lecithin hybrid nanoparticle
Cyt c	cytochrome c
DAU	daunorubicin
DGL	dendrigraftpoly-L-lysines
DOTAP	1,2-dioleoyl-3-trimethylammonium-propane
DNR	daunorubicin
DOX	doxorubicin
dsDNA	double-stranded DNA
DSPE	1,2-distearoyl-sn-glycero-3-phosphoethanolamine
EGFR	Epidermal growth factor receptor
EHH	electrostatic adsorption, hydrogen bonding, and hydrophobic interaction
Ehrlich’s ACC	Ehrlich’s ascites carcinoma cell
ELP	elastin-like polypeptide
EpCAM	epithelial cell adhesion molecule
EPI	epirubicin
FGFR1	fibroblast growth factor receptor type-1
FMSN	fluorescent mesoporous silica nanoparticle
FN	fibronectin
FO	Ferric oxide
FoxM1	Forkhead box M1
Gd:SrHap	gadolinium-doped luminescent and mesoporous strontium hydroxyapatite
GMNP	gold-coated magnetic nanoparticle
GNP	gold nanoparticle
GO	Graphene oxide
GPN	gefitinib-loaded poly (lactic co-glycolic acid) nanoparticle
GQD	graphene quantum dot
GST	glutathione S-transferase
HA	Hyaluronic acid
HAS-CS	human serum albumin coated with chitosan
HBLL	human B cell leukemia and lymphoma
HCC	Hepatocellular carcinoma
HER3	human epidermal growth factor receptor 3
His	hexahistidine
HMME	is a photosensitizer
HPA	heparanase
HPAEG	poly(2-((2-(acryloyloxy)ethyl)disulfanyl)ethyl 4-cyano-4-(((propylthio)carbonothioyl)-thio)-pentanoate-co-poly(ethylene glycol) methacrylate)
HSP71	heat shock cognate 71 kDa protein
HTT	hyperthermia therapy
IL-6R	interleukin-6 receptor
IONP	Iron oxide nanoparticle
KG6E	glutamic acid-modified dendritic poly(L-lysine) system
KLA	(KLAKLAK)_2_ peptide
LP-DNA	liposome-polycation-DNA
MAA	methacrylamide
MAGE	melanoma-associated peptide antigen
MAL	maleimide
MASI	N-(methacryloxy)succinimide
MCS	Myristylated Chitosan
MMA	methyl methacrylate
MOF	(mesoporous) metal-organic framework
MPC	mesoporous carbon
MPEG	Poly(ethylene glycol) methyl ether
M-PLGA	mannitol-functionalized poly(lactide-co-glycolide)
MSN	Mesoporous silica nanoparticle
mTEC	mouse tumor endothelial cell
MDR	multiple drug resistance
MUC1	Mucin-1
NHS	N-hydroxysuccinimide
NIR PLN	near infrared-persistent luminescence nanomaterials
NMOF	amino-triphenyl dicarboxylate-bridged Zr^4+^ metal-organic framework nanoparticle
NP	nanoparticle
NSCLC	non-small cell lung cancer
ONT	oligonucleotide
PAA	polyacrylic acid
PAM	Peptide amphiphile micelle
PAMAM	polyamidoamine
PBABT	poly (butylene adipate-co-butylene terephthalate)
PCL	poly(ε-capro-lactone)
PDA	hydrochloride dopamine
PDGFR	platelet-derived growth factor receptor
PEC	polyelectrolyte complexe
PEEUA	polyethylenimine-urocanic acid
PEG	polyethylene glycol
PEI	polyethylene imine
PF127	Pluronic F127
PFK15	1-(4-pyridyl)-3-(2-quinoline)-2-propyl-1-one (an aerobic glycolysis inhibitor)
PFOB	Perfluorooctylbromide
PGFRβ	platelet-derived growth factor receptor β
P-gp	P-glycoprotein
PLA	poly(lactic acid)
PLGA	poly(lactic-co-glycolic acid)
PLK1	Polo-Like Kinase 1
PLL	poly (L-lysine)
pPEGMA-PCL-pPEGMA	poly(poly(ethylene glycol) methacrylate)-poly(caprolactone)-poly(poly(ethylene glycol) methacrylate)
PTK7	protein tyrosine kinase-7
PTT	Photothermal therapy
PVP	poly (N-vinylpyrrolidone)
PβAE	poly (β amino ester)
QD	quantum dot
RBCm	red blood cell membrane
SATB1	special AT-rich sequence binding protein 1
SPION	superparamagnetic iron oxide nanoparticles
SPMFN	Superparamagnetic Ferroarabinogalactan Nanoparticles
TAG-72	tumor-associated glycoprotein 72
TD	thiolated dextran
TiO_2_	titanium dioxide
TLR	Toll-like receptor TLR4-siRNA
TM-JM1/2	transmembrane-juxtamembrane 1/2 domain
TMPyP	5, 10, 15, 20-tetra (phenyl-4-N-methyl-4-pyridyl) porphyrin
TMPyP4	5,10,15,20-tetrakis(1-methylpyridinium-4-yl) porphyrin
TNBC	triple-negative breast cancer
TPGS	D-α-tocopheryl polyethylene glycol 1000 succinate
TSP	thermosensitive polymer
UCNP	up-conversion luminescent
NaYF4	Yb(3+)/Er(3+) nanoparticle
VEGF	vascular endothelial growth factor
β-CD	β-cyclodextrin
